# The Diversity Patterns of Rare to Abundant Microbial Eukaryotes Across a Broad Range of Salinities in a Solar Saltern

**DOI:** 10.1007/s00248-021-01918-1

**Published:** 2021-11-15

**Authors:** Hyeon Been Lee, Dong Hyuk Jeong, Byung Cheol Cho, Jong Soo Park

**Affiliations:** 1 Department of Oceanography, Kyungpook National University, Daegu, 41566 Republic of Korea; 2 School of Earth and Environmental Sciences, Seoul National University, Seoul, 08826 Republic of Korea; 3 Saemangeum Environmental Research Center, Kunsan National University, Kunsan, 54150 Republic of Korea

**Keywords:** Microbial eukaryotic diversity, Hypersaline ecosystems, Solar saltern, Salinity gradients, Operational taxonomic units

## Abstract

Solar salterns are excellent artificial systems for examining species diversity and succession along salinity gradients. Here, the eukaryotic community in surface water of a Korean solar saltern (30 to 380 practical salinity units) was investigated from April 2019 to October 2020 using Illumina sequencing targeting the V4 and V9 regions of 18S rDNA. A total of 926 operational taxonomic units (OTUs) and 1,999 OTUs were obtained with the V4 and V9 regions, respectively. Notably, most of the OTUs were microbial eukaryotes, and the high-abundance groups (> 5% relative abundance (RA), Alveolata, Stramenopila, Archaeplastida, and Opisthokonta) usually accounted for > 90% of the total cumulative read counts and > 80% of all OTUs. Moreover, the high-abundance Alveolata (larger forms) and Stramenopila (smaller forms) groups displayed a significant inverse relationship, probably due to predator–prey interactions. Most of the low-abundance (0.1–5% RA) and rare (< 0.1% RA) groups remained small portion during the field surveys. Taxonomic novelty (at < 90% sequence identity) was high in the Amoebozoa, Cryptista, Haptista, Rhizaria, and Stramenopila groups (69.8% of all novel OTUs), suggesting the presence of a large number of hidden species in hypersaline environments. Remarkably, the high-abundance groups had little overlap with the other groups, implying the weakness of rare-to-prevalent community dynamics. The low-abundance Discoba group alone temporarily became the high-abundance group, suggesting that it is an opportunistic group. Overall, the composition and diversity of the eukaryotic community in hypersaline environments may be persistently stabilized, despite diverse disturbance events.

## Introduction

Hypersaline environments (> 40 practical salinity units, psu), which are globally distributed but sparsely localized, are home to communities of halophilic and halotolerant microorganisms, including both prokaryotes and eukaryotes [1–3]. Artificial solar salterns are very attractive systems for analyzing biodiversity patterns over a broad range of salinities since they include many water bodies with very different mineral contents and biological activities within walking distance of each other [2, 4, 5]. Halophilic or halotolerant prokaryotes have been extensively examined [3, 6, 7], whereas studies of eukaryotes in hypersaline environments have lagged behind those of prokaryotes, in part due to the lower abundance (or absence) of eukaryotes in many of these systems [8, 9]. Among eukaryotes, the primary producer *Dunaliella* (a chlorophyte) and the zooplankton crustacean *Artemia* (brine shrimp) have had their niches commonly described in hypersaline ecosystems, but our knowledge of most other eukaryotes is much more restricted [2, 9]. This has likely contributed to the view that the microbial food web in hypersaline systems is much simpler than in marine and freshwater systems [4, 8, 9].

Autotrophic halophilic eukaryotes, *Dunaliella* spp., and fungi have been frequently detected with cultivation and microscopy approaches [2, 10], whereas protozoan species in hypersaline environments have rarely been reported. Since the 2000s, various heterotrophic microbial eukaryotes have been successfully cultured from hypersaline environments and then identified and classified by morphology, ultrastructure, and molecular phylogenies based on 18S rRNA gene sequencing [1]. Most of them were regarded as bacterivores based on their feeding behavior under cultivation, although some isolates (e.g., the ciliate *Trimyema koreanum*) were shown to be capable of feeding on *Dunaliella*, potentially revealing more trophic complexity in hypersaline systems than previously thought [4, 11]. Additionally, some obligate predators of other eukaryotes are known to exist (e.g., *Colpodella* and *Palustrimonas*), even though they have not been isolated under stable predator–prey culture [12, 13]. Heterotrophic isolates from hypersaline environments were assigned to Heterolobosea in Discoba (*Euplaesiobystra*, *Pharyngomonas*, *Pleurostomum*, *Tulamoeba*, *Selenaion*, *Percolomonas*, and *Aurem*), Ciliophora in Alveolata (*Trimyema*, *Fabrea*, *Schmidingerothrix*, and *Platynematum*), Stramenopila (*Aladia*, *Halocafeteria,* and *Haloplacidia*), *Colpodella* in Alveolata, and *Palustrimonas* in Alveolata [1, 14–17]. Many of these genera are restricted to hypersaline environments, proved to be mostly novel species in these systems. The diversity within hypersaline habitats is substantial as well. Park et al. [18] noted that more than 25 different protozoan morphospecies had been recorded in previous studies on saturated or nearly saturated brines (> 300 psu). Consistent with this pattern, culture-independent environmental sequencing studies have also revealed the high novelty level of microbial eukaryotes along salinity gradients in solar salterns [19, 20].

Many halophilic or halotolerant microbial eukaryotes (e.g., *Halocafeteria*, *Pleurostomum*, *Trimyema*, *Euplaesiobystra*, *Tulamoeba*, and *Aurem*) were discovered in Korean solar salterns, suggesting that a diverse microbial eukaryote is present in these habitats [11, 12, 18, 21–24]. However, the overall biodiversity of the high-abundance (> 5% relative abundance (RA)) and rare (< 0.1% RA) [25] eukaryotic groups in Korean solar salterns has not yet been examined using the next-generation sequencing (NGS) approach, which has revealed extensive microbial eukaryote diversity in other systems [19, 26, 27]. The rare biosphere taxa are regarded as low-abundance groups with cut-offs of 0.1% or 0.01% in sequencing counts and have critical ecological roles over time in natural ecosystems, acting as a seed bank that can become abundant under favorable environmental conditions [25]. Remarkably, almost nothing is known about the diversity and temporal profiles of abundant and rare eukaryotic groups along salinity gradients across different seasons and years. Furthermore, the comparison of culture-dependent and culture-independent surveys for the hypersaline biota remains poorly understood. As a result, the inventory of the eukaryotic community in hypersaline environments is still insufficient. Based on the NGS approach, the V4 and V9 regions of 18S rDNA had been commonly used for the diversity and molecular phylogeny of eukaryotes [26, 28, 29]. The combination of the V4 and V9 regions sequencing data was more advantageous for assessing the diversity and evolutionary relationship of eukaryotes than data from one biomarker [26]. Thus, V4 and V9 sequence data obtained with NGS are capable of providing important information on the diversity, ecology, and evolutionary history of the hypersaline biota. Here, we investigated eukaryotic communities in the Eui-Seong solar saltern from 30 to 380 practical salinity units (psu) in April, June, and August 2019 and October 2020. Using the Illumina MiSeq platform, we targeted the V4 and V9 regions of 18S rDNA and compared the NGS data with previous data in GenBank on eukaryotes from hypersaline environments. We studied the diversity and distribution of eukaryotes (mostly protists) along a salinity gradient in the field surveys to test the following hypothesis: 1) The diversity of eukaryotes decreases with increasing salinity. 2) the degree of eukaryotic novelty is relatively high in this unique ecosystem. 3) The NGS data are distinct from results in previous studies. 4) Besides abiotic factors (e.g. salinity and temperature), another factor can influence the diversity and distribution of eukaryotes. Overall, we provide information on the RA, distribution, and novelty patterns of eukaryotes along a salinity gradient across different seasons and years.

## Materials and Methods

### Sampling Site and Sample Collection

**Fig. 1:**
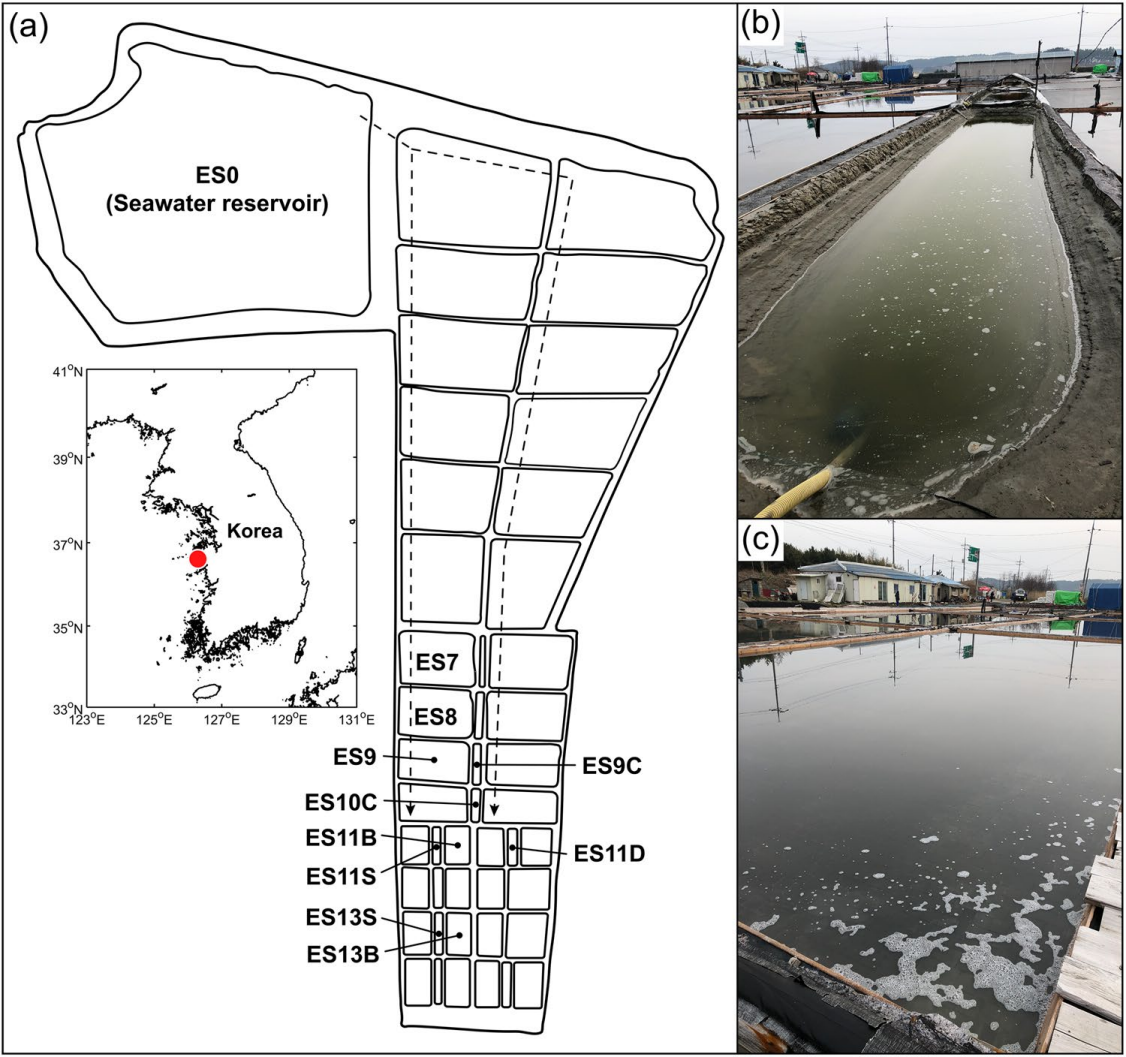
The sampling location showing ponds in the Eui-Seong (ES) solar saltern (red circle), Republic of Korea. (**a**) Map of the ES solar saltern. Dotted line arrows represent the water flow direction from normal seawater (ES0). (**b**) Brine storage in the ES solar saltern. (**c**) Crystallization pond in the ES solar saltern

A Eui-Seong solar saltern (ES, thalassic, total area: 43,818 m^2^) located in Taean on the west coast of the Republic of Korea (36°36′08.3"N 126°17′49.3"E, Fig. 1) consists of a series of ponds with salinity ranging from that of ordinary seawater (ES0, area: 12,578 m^2^) to NaCl saturation. Twelve surface waters with different salinities were collected from the saltern edge of eleven ponds using 500 mL HDPE bottles (DH.B03114, Daihan Scientific Co., Ltd., Republic of Korea) presoaked in 10% (v/v) HCl. Samples were collected in April, June, and August 2019 and October 2020 when the day light was plenty. The salinity, pH, and temperature of the samples were measured using a handheld refractometer (MASTER-S28α, ATAGO Co., Ltd., Tokyo, Japan), pH meter (EcoTestr pH1, Eutech Instruments, Thermo Fisher Scientific, Waltham, MA, USA), and thermometer (CENTER 300, CENTER Technology Corp., Taipei, Taiwan), respectively. Chlorophyll-*a* concentrations were measured by taking surface water samples (55–500 mL). After filtering the samples onto a glass microfiber filter (GF5 grade, 47 mm, CHMLAB Group, Barcelona, Spain), the extraction of chlorophyll-*a* was performed in duplicate as described in Parsons et al. [30].

### Nucleic Acid Extraction

The surface water samples (300–3,100 mL) were prefiltered through a 200-μm-pore-size testing sieve to remove large debris. Subsequently, the prefiltered subsamples were collected on several 0.45- or 0.65-μm-pore-size PVDF membrane filters (47 mm, Durapore®, Merck Millipore, Billerica, MA, USA) using a vacuum pump (DOA-P704-AC, Gast Manufacturing Inc., Benton Harbor, MI, USA). These filters were stored in a 50-mL conical tube at –20 °C and were moved to the laboratory for further analyses. For environmental DNA extraction, the filters were sliced into small pieces, and 1 mg mL^−1^ lysozyme (final concentration; L6876, Sigma-Aldrich, St. Louis, MO, USA) was added. The tubes were then incubated at 37 °C for 30 min, and then 0.4 mg mL^−1^ proteinase K (final concentration, Biosesang™, Seongnam, Republic of Korea) and 1% (w/v) sodium dodecyl sulfate (final concentration, Bioneer, Daejeon, Korea) were added. The tubes were then incubated at 55 °C for 2 h. Nucleic acids were further purified using 50 μg mL^–1^ GlycoBlue™ Coprecipitant (final concentration, Invitrogen™, Thermo Fisher Scientific, Waltham, MA, USA) and a DNeasy Blood and Tissue Kit (Qiagen, Hilden, Germany) as described in the manufacturer’s instructions. The extracted DNA concentrations were measured with a Quantus™ fluorometer and QuantiFluor® ONE dsDNA System (Promega Corp., Madison, WI, USA), with 0.72–47.13 ng μL^−1^ extracted DNA obtained.

### Illumina Sequencing

Two different primer sets that amplified portions of the 18S rRNA gene were used for Illumina sequencing. The V4 forward (5′-CCAGCAGCCGCGGTAATTCC-3′) and V4 reverse (5′-ACTTTCGTTCTTGATTAA-3′) primers were used to target the V4 hypervariable region [26, 31], and the V9 forward (5′-CCCTGCCHTTTGTACACAC-3′) and V9 reverse (5′-CCTTCYGCAGGTTCACCTAC-3′) primers were used to target the V9 hypervariable region [26, 32]. The PCR steps for the V4 regions comprised an initial denaturing step at 95 °C for 5 min, followed by 10 cycles of 94 °C for 30 s, 57 °C for 45 s, 72 °C for 1 min, and then 15 cycles of 94 °C for 30 s, 47 °C for 45 s, and 72 °C for 1 min, followed by a final hold at 72 °C for 10 min [26, 33]. Amplification conditions for the V9 region comprised an initial denaturing step at 94 °C for 3 min, followed by 30 cycles of 94 °C for 30 s, 57 °C for 60 s, and 72 °C for 90 s and a final hold at 72 °C for 10 min [26, 32]. The library was quantified using qPCR, as described in the Illumina qPCR quantification protocol guide, and sequenced using the Illumina MiSeq Reagent Kit v3 (Illumina Inc., San Diego, CA, USA) at Macrogen Inc., Seoul, Republic of Korea.

### Sequence Analysis and Phylogenetic Analysis

Paired-end reads were merged by Fast Length Adjustment of SHort reads 1.2.11 (FLASH) [34]. Size selection and trimming of reads were conducted using CD-HIT-OTU software (v.0.0.1 for Illumina rRNA data) [35]. Through this software, chimeric and noise sequences were removed. The filtered sequences were clustered with a 97% identity threshold and assigned to OTUs according to previous studies [26, 36–39]. Taxonomic classification of sequences was conducted with QIIME UCLUST [40], and the National Center for Biotechnology Information database was used as the reference data for the 18S rRNA gene sequences [26]. Rarefaction analysis was performed with DataGraph 4.6 to assess sampling sufficiency and compare species richness between subsamples [41].

OTUs were assigned to eukaryotic groups, broadly following recent summaries of eukaryote diversity [42, 43], as follows: Archaeplastida, CRuMs, Cryptista, Discoba, Haptista, Telonemia, Stramenopila, Alveolata, Rhizaria, Amoebozoa, Apusomonada, Breviates, Opisthokonta, Ancyromonadida and Picozoa. Other major eukaryotic taxa (e.g., Metamonada) were not detected.

We constructed three groups of datasets for phylogenetic analysis: Heterolobosea, Stramenopila, and Alveolata. These groups were thought to include protozoan species frequently isolated from hypersaline environments: 1) The 18S rRNA gene sequences from 94 representative heterolobosean species, including 31 sequences of the V9 region (lack of sequences in the V4 region) and 16 representative nonheterolobosean species as an outgroup (the seed alignment originated from Tikhonenkov et al.) [17], 2) Two datasets containing i) 67 representative Stramenopila sequences of the 18S rRNA gene and 41 Stramenopila sequences of the V4 region (Stramenopila + V4) and ii) 67 representative Stramenopila sequences of the 18S rRNA gene and 98 Stramenopila sequences of the V9 region (Stramenopila + V9) (the seed alignment originated from Park and Simpson) [44], and 3) Two datasets, i) 160 representative Alveolata sequences of the 18S rRNA gene and 105 Alveolata sequences of the V4 region (Alveolata + V4) and ii) 160 representative Aveolata sequences of the 18S rRNA gene and 186 Alveolata sequences of the V9 region (Alveolata + V9) (the seed alignment originated from Park and Simpson) [12]. Eight representative Stramenopila species were used as outgroups in the two datasets. The datasets were aligned using MAFFT v.7 [45] and then masked by eye. Maximum likelihood trees were estimated using IQ-tree 1.6.12. The GTR + F + I + G4 model for Heterolobosea, TN + F + I + G4 model for Stramenopila + V4, and TIM2 + F + I + G4 model for Stramenopila + V9, Alveolata + V4, and Alveolata + V9 were selected through the best-fit model test option (-m TEST) [46–48]. Statistical support was assessed using bootstrapping with 1,000 replicates.

### Statistical Analysis

The Shapiro–Wilk test, a nonparametric statistical test, was performed to assess the normality of the dataset for regression analysis. Levene’s test was used to check for homogeneity of variances. To reveal statistically significant differences, *T*-tests and Mann–Whitney *U* tests were performed for normally and nonnormally distributed data, respectively. All statistical analyses were performed using SPSS v25.0.

## Results

### Characterization of the Eui-Seong Solar Saltern

The salinity of the 12 surface water samples obtained from 11 salt pans in the Eui-Seong solar saltern ranged between 30 and 380 psu (Fig. 1; Table 1). The water temperature of the samples varied seasonally between 14.5 °C and 42.5 °C. In summer (June and August), it was generally higher than in spring (April) and fall (October). The pH value of the samples ranged between 6.9 and 8.9. The concentration of chlorophyll-*a* ranged between < 0.1 and 16.0 μg L^–1^ (Table 1).

**Table 1:** Environmental parameters for the twelve samples of the Eui-Seong solar saltern, Republic of Korea

Sample ID	Date (day/month/year)	Site	Area (m^2^)	Salinity (psu)	Temperature (°C)	pH	Chlorophyll-*a* (μg L^–1^)
168 psu, Apr 19	12/April/2019	ES11B	164	168	26.2	6.9	0.9
208 psu, Apr 19	12/April/2019	ES11D	53	208	27.0	6.9	< 0.1
248 psu, Jun 19	05/June/2019	ES11S	33	248	29.3	7.9	11.4
300 psu, Jun 19	05/June/2019	ES8	595	300	29.4	7.6	3.8
76 psu, Aug 19	26/August/2019	ES7	674	76	37.9	8.9	0.3
124 psu, Aug 19	26/August/2019	ES9	502	124	42.5	8.5	1.4
180 psu, Aug 19	26/August/2019	ES10C	25	180	33.6	7.9	0.1
30 psu, Oct 20	13/October/2020	ES0	12,578	30	16.4	8.9	14.7
78 psu, Oct 20	13/October/2020	ES8	595	78	14.5	8.7	4.1
134 psu, Oct 20	13/October/2020	ES9C	39	134	16.3	8.3	16.0
248 psu, Oct 20	13/October/2020	ES13S	48	248	15.5	7.7	2.9
380 psu, Oct 20	13/October/2020	ES13B	178	380	16.8	7.2	3.1

### Illumina Sequencing and Abundant and Rare Eukaryotic Groups

**Fig. 2:**
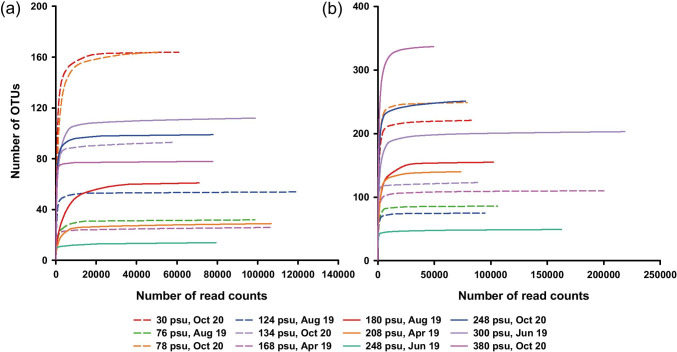
Rarefaction curves of the V4 (**a**) and V9 (**b**) operational taxonomic units (OTUs; 97% sequence identity threshold) from the different samples (practical salinity units: psu)

Illumina sequencing was performed on the 12 samples using two different barcode regions: V4 and V9. A total of 1,466,403 V4 reads and 1,793,983 V9 reads were obtained (Table 2). After filtering ambiguous, low-quality (quality score offset of 33), chimeric, and short (less than 36 bp) sequences, our dataset contained total read counts of 1,016,659 and 1,368,001 for the V4 and V9 regions of 18S rDNA, respectively (Table 2). The average amplicon sizes for the V4 and V9 sequences were 363 bp (± 21 bp, standard deviation) and 138 bp (± 11 bp), respectively. The numbers of eukaryotic operational taxonomic units (OTUs) at a 97% identity threshold were 926 and 1,999 for the V4 and V9 regions, respectively (Table 2). In the present study, rarefaction curves suggested that OTUs in both the V4 and V9 datasets were sufficiently determined (Fig. 2).

**Table 2:** Summary of Illumina sequence data from the V4 and V9 regions of 18S rDNA

Sequence description	V4	V9
Amplicon size	363 (± 21)	138 (± 11)
Total bases	604,114,600	299,538,799
Read count	1,466,403	1,793,983
Filtered read count	1,016,659	1,368,001
Ambiguous	48	12
Wrong prefix of primers	88,131	37,374
Low-quality	1,108	0
Chimera	26,461	29,510
Other (non-sequencing error)	333,996	359,086
OTUs (Eukaryotic reads)	926	1,999

Amplicon size, mean (± std)

Ambiguous, filtered sequences with ambiguous base calls; Low-quality, filtered sequences with low-quality bases (Quality score offset 33); Other (non-sequencing error), query coverage and identity percentage with < 85%

The OTUs based on the V4 and V9 regions were mostly microbial eukaryotes and assigned to the following fifteen eukaryotic groups: Alveolata, Archaeplastida, Opisthokonta, Stramenopila, CRuMs, Cryptista, Discoba, Haptista, Amoebozoa, Ancyromonadida, Apusomonada, Breviates, Picozoa, Rhizaria, and Telonemia [42, 43]. Furthermore, we classified the fifteen groups into three subgroups based on the RA of the read counts in hypersaline environments: rare group (RA of < 0.1%), low-abundance group (RA of 0.1–5%), and high-abundance group (RA of > 5%).

**Fig. 3:**
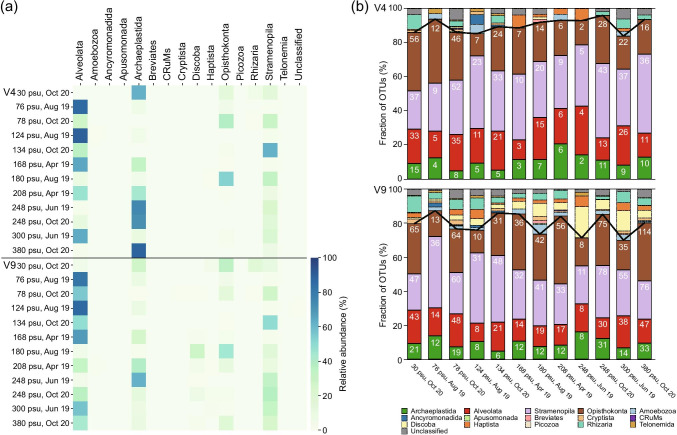
Relative abundance (RA) and fraction of operational taxonomic units (OTUs). (**a**) Heatmap of the relative abundance of the V4 and V9 sequences along the salinity gradient. (**b**) Bar chart of fraction of OTUs of the V4 and V9 datasets along the salinity gradient. The numerical values in the bar chart represent the number of OTUs within each group. Solid lines represent the cumulative proportions of the four high-abundance groups (RA of > 5%, Archaeplastida, Alveolata, Stramenopila, and Opisthokonta) in the hypersaline environments

**Fig. 4:**
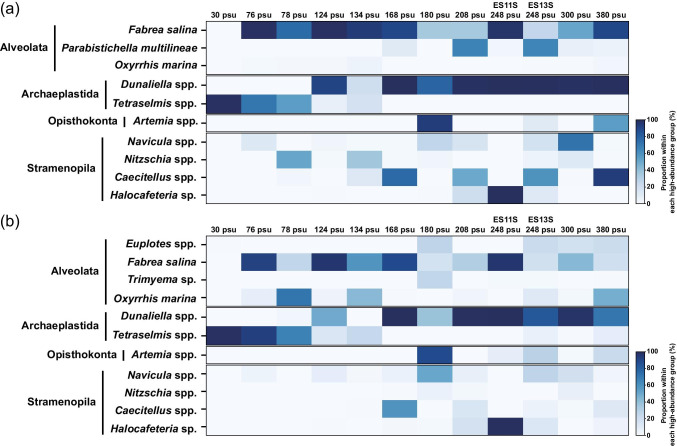
The distribution patterns of the major species within the high-abundance groups Alveolata, Archaeplastida, Opisthokonta, and Stramenopila along the salinity gradient (practical salinity units: psu) based on the read counts in the V4 (**a**) and V9 (**b**) datasets (for each sampling site, see Table 1 and Fig. 1). The major species was determined up to the fourth most abundant species (> 5% relative abundance) detected at > 100 psu

Based on the read counts, Alveolata, Archaeplastida, Opisthokonta, and Stramenopila were generally abundant in the classified eukaryotic groups in hypersaline and ordinary seawater (i.e. 30 psu) environments, suggesting they were high-abundance groups (Fig. 3a). Alveolata was the most abundant taxon overall and was especially dominant in the 76, 124, 168, and 300 psu samples, comprising 63.4–89.3% (average of 75.9%) and 56.8–87.0% (average of 73.3%) of the V4 and V9 sequences, respectively (Fig. 3a). More than 90% of Alveolata reads were assigned to Ciliophora, particularly the halotolerant ciliate *Fabrea salina* (Fig. 4). For both the V4 and V9 regions at 248 and 380 psu, Archaeplastida was often the most abundant, composed mainly of *Dunaliella* spp. (99–100% and 71–100% in the V4 and V9 regions, respectively), which are halotolerant chlorophytes (Fig. 4). Opisthokonta reads were predominant at 180 psu for both the V4 and V9 regions (Fig. 3a). Most of the Opisthokonta reads (93.3% for V4; 87.6% for V9) were assigned to the brine shrimp (*Artemia*) group (Fig. 4). The Stramenopila group had a high RA in the 134 and 248 psu samples (Fig. 3a). Within Stramenopila, the class Bacillariophyta (diatoms) was dominant at 134 psu, while the halophilic bicosoecid *Halocafeteria* was the dominant species at 248 psu (Fig. 4). Amoebozoa, Apusomonada, Breviates, Rhizaria, Telonemia, Ancyromonadida, CRuMs, Cryptista, Haptista, Discoba and Picozoa either showed relatively low read counts for both the V4 and V9 regions or were sometimes undetected by primer sets used in the hypersaline environments, suggesting they were low-abundance (Amoebozoa, Rhizaria, Telonemia, Cryptista, Discoba, and Haptista) or rare (Ancyromonadida, Breviates, Apusomonada, CRuMs, and Picozoa) groups (Fig. 3a). Discoba was detected in the V9 region dataset (up to 28.2% at 180 psu in August 2019) but was rarely found in the V4 region dataset (Fig. 3a). Most (> 90%) Discoba sequences were related to Heterolobosea (not shown). Rhizaria, Cryptista, and Haptista sequences were found more in the V9 region dataset than in the V4 region dataset (Fig. 3a). Unclassified species not assigned to any group were also detected and were more abundant in the V9 biomarker dataset than in the V4 biomarker dataset (Fig. 3a).

**Fig. 5:**
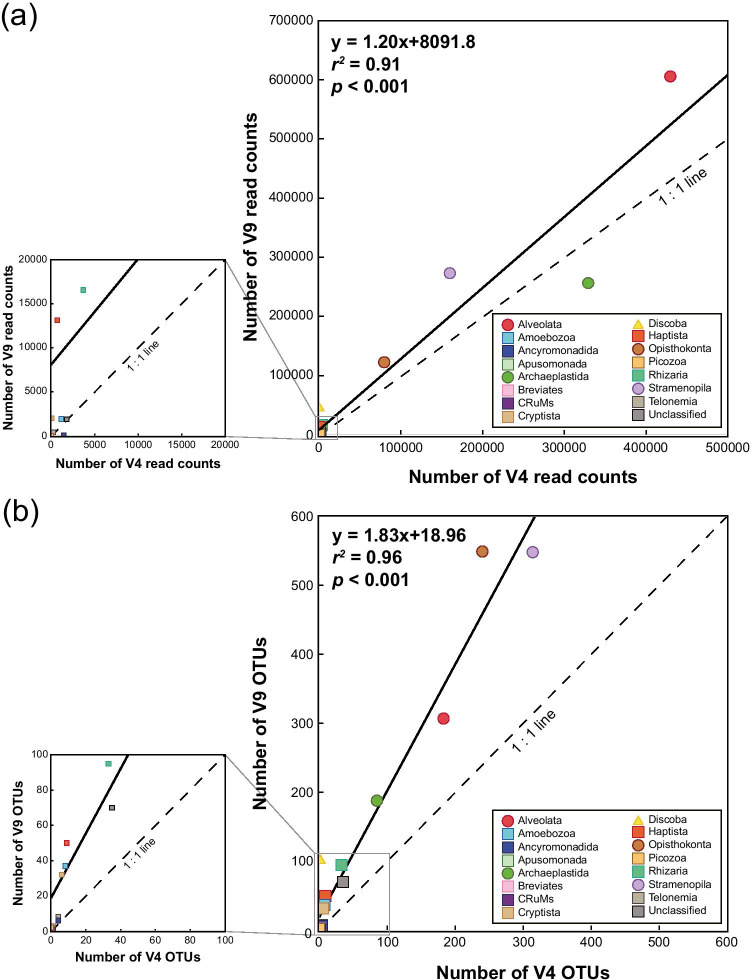
Relationships between the V4 and V9 regions of 18S rDNA of eukaryotic groups based on read counts (**a**) and OTUs (**b**)

**Fig. 6:**
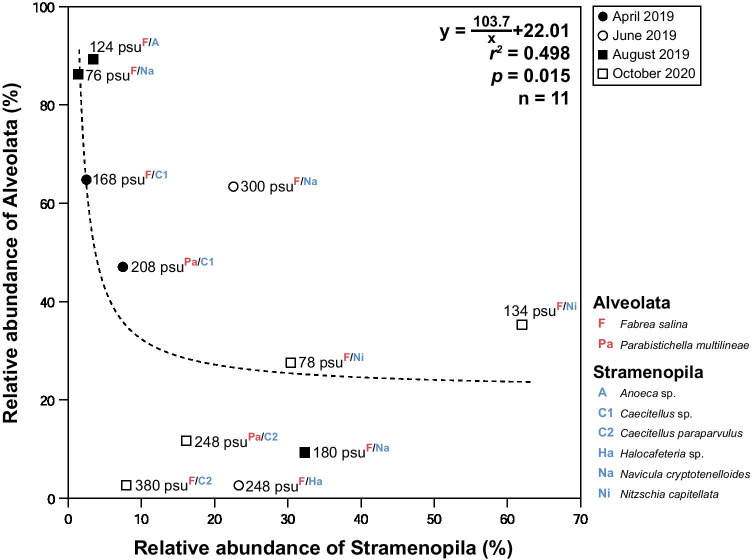
Relationship between the relative abundances of Alveolata and Stramenopila based on the read counts of the V4 dataset. The red and blue annotations represent the most abundant species in Alveolata and Stramenopila, respectively

Alveolata (3–35 OTUs for V4; 8–48 OTUs for V9), Archaeplastida (2–11 OTUs for V4; 6–33 OTUs for V9), Opisthokonta (2–46 OTUs for V4; 8–114 OTUs for V9), and Stramenopila (5–52 OTUs for V4; 11–78 OTUs for V9) also dominated the OTU data from 76 to 380 psu in the hypersaline environments (> 40 psu, Fig. 3b), accounting for 84–96% and 70–87% of the total OTUs in the V4 and V9 region datasets, respectively (Fig. 3b). The V9 dataset showed significantly higher eukaryotic read counts (*r*^*2*^ = 0.91, *p* < 0.001) and OTU abundances (*r*^*2*^ = 0.96, *p* < 0.001) than the V4 dataset in the present study (Fig. 5). Notably, in the V4 dataset, there was a significant inverse relationship between Alveolata and Stramenopila based on the RA (*r*^*2*^ = 0.498, *p* = 0.015; Fig. 6), but the relationship was not significant in the V9 dataset (*p* = 0.151, Mann–Whitney U test).

### Phylotype Novelty in Hypersaline Environments

Based on a BLASTn search, the proportion of OTUs with less than 90% identity with the closest sequence in GenBank was 7.3% and 14.6% of all OTUs (891 OTUs for V4 and 1929 OTUs for V9) in the V4 and V9 region datasets, respectively, consisting of fifteen taxon groups excluding the unclassified group (Table 3). This ‘eukaryote novelty’ appeared to be the highest at > 150 psu in the V9 region dataset in April (169 psu and 208 psu), June (248 psu and 300 psu) and August (180 psu) 2019 and October (248 psu and 380 psu) 2020. The highest number of OTUs with less than 90% identity belonged to the high-abundance group Stramenopila (V4: 35 OTUs, V9: 115 OTUs) and the low-abundance group Rhizaria (V4: 11 OTUs, V9: 44 OTUs) (Table 3). Within each high-abundance group, the level of novelty was relatively low: Alveolata (6.0% for V4, 2.9% for V9), Archaeplaestida (0% for V4, 8.0% for V9), Opisthokonta (1.7% for V4, 8.4% for V9), and Stramenopila (11.1% for V4, 21.0% for V9) (Table 3). In the rare and low-abundance groups (each representing < 3% of all OTUs), the level of novelty could be as high as 100% (Table 3).

**Table 3:** The number of operational taxonomic units (OTUs) in diverse eukaryote groups and the proportion of OTUs with less than 90% identity with the closest sequence in GenBank in the V4 and V9 region datasets

Taxon groups	Total OTUs		OTUs (< 90% identity)	
	V4	V9	V4	V9
Alveolata	183	307	11 (6.0%)	9 (2.9%)
Amoebozoa	8	37	1 (12.5%)	8 (21.6%)
Ancyromonadida	4	6	nd	nd
Apusomonada	1	2	nd	2 (100%)
Archaeplastida	85	188	nd	15 (8.0%)
Breviates	1	3	1 (100%)	nd
CRuMs	1	1	1 (100%)	nd
Cryptista	6	32	nd	11 (34.4%)
Discoba	2	101	1 (50%)	8 (7.9%)
Haptista	9	50	nd	18 (36.0%)
Opisthokonta	240	549	4 (1.7%)	46 (8.4%)
Picozoa	nd	2	nd	nd
Rhizaria	33	95	11 (33.3%)	44 (46.3%)
Stramenopila	314	548	35 (11.1%)	115 (21.0%)
Telonemia	4	8	nd	5 (62.5%)
Total	891	1,929	65 (7.3%)	281 (14.6%)

*nd*, not detected

### Phylogenetic Comparison with Previously Obtained Culture-Based Isolates

**Fig. 7:**
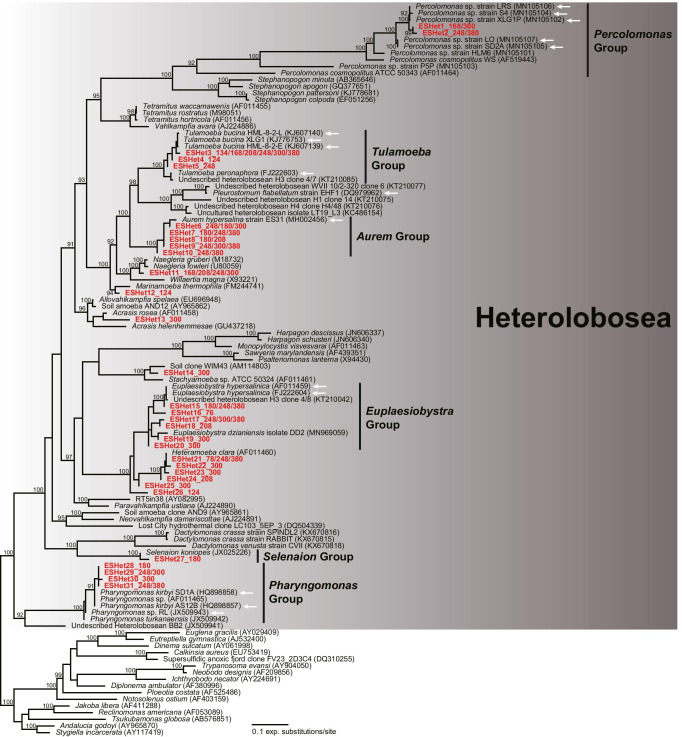
Maximum likelihood phylogenetic tree of 18S rDNA sequences from heterolobosean species. The sequence name from the V9 region dataset alone is marked in red, and the numerical values after the sequence name indicate the practical salinity units of the samples in which each OTU was found. White arrows represent halophilic species, which can grow best at 75 psu or higher. Bootstrap support values are shown at the nodes when > 90%

Previous studies based on culture-dependent approaches suggested Heterolobosea, Stramenopila, Archaeplastida, and Alveolata as the predominant eukaryotic groups in a variety of hypersaline environments [1, 2, 11–13, 17, 18, 22, 24, 44, 49–52]. Heterolobosea includes many described halophilic/halotolerant eukaryotic species [1, 17, 18, 22, 24, 52]. Here, environmental sequences belonging to Heterolobosea (part of Discoba) were present in the V9 dataset but were nearly absent from the V4 dataset. Most Heterolobosea-affiliated V9 sequences were on branches with previously identified halophilic/halotolerant heterolobosean species (e.g., *Percolomonas*, *Tulamoeba*, *Aurem*, *Euplaesiobystra*, *Selenaion*, and *Pharyngomonas*) with high bootstrap support (ML: 74–100%, Fig. 7). A few sequences obtained at > 150 psu were related to marine or freshwater/soil species instead (Fig. 7, e.g., *Naegleria* and *Heteramoeba*).

**Fig. 8:**
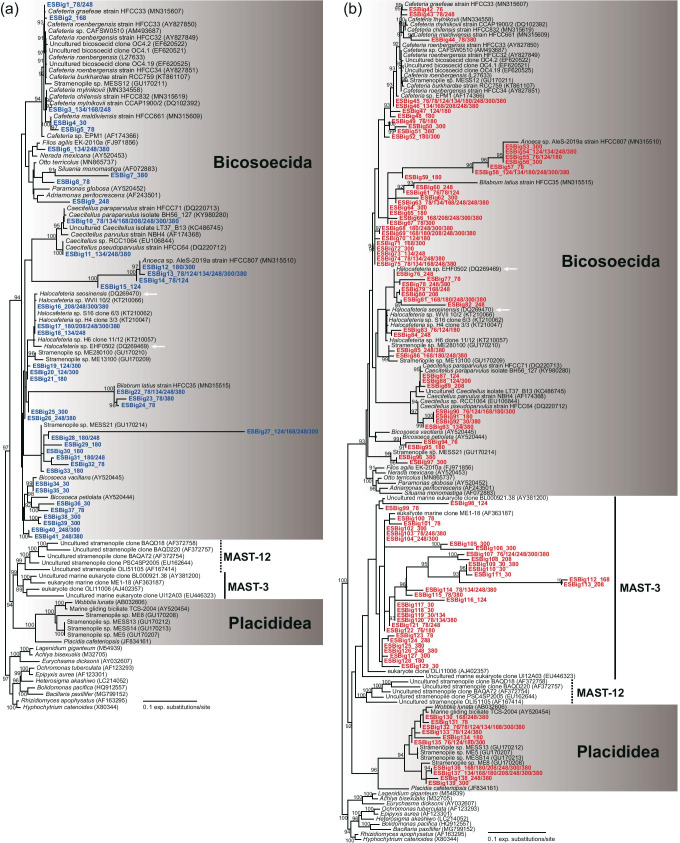
Maximum likelihood phylogenetic tree of the V4 (**a**) and V9 (**b**) regions of 18S rDNA sequences from Stramenopila. The sequence names from the V4 and V9 region datasets are marked in blue and red, respectively. The numerical values after the sequence name indicate the practical salinity units of the samples in which each OTU was found. White arrows represent halophilic species, which can grow best at 75 psu or higher. Bootstrap support values are shown at the nodes when > 90%. MAST indicates marine stramenopiles

The Bicosoecida was the taxon group of stramenopiles with the greatest number of OTUs in the V4 region dataset (ML: 94%, Fig. 8a). This included OTUs belonging to the known halophilic group *Halocafeteria* but also relatively abundant/widespread OTUs related to *Anoeca* and *Caecitellus*, among others. No Placididea group sequences were found, even though this group includes previously isolated halotolerant species [44]. Conversely, the V9 region dataset sequences were distributed among diverse groups, including Bicosoecida, Placididea, and MASTs, with high bootstrap support (ML: 90–96%, Fig. 8b). Several sequences from the V9 region dataset were mainly assigned to Bicosoecida, Placididea and to presumable relatives of the MAST-3 group of uncultured stramenopiles (Fig. 8b). Furthermore, the V9 sequences within the Placididea (ML: 96%) were distantly related to those of cultivated halotolerant species (Fig. 8b).

**Fig. 9:**
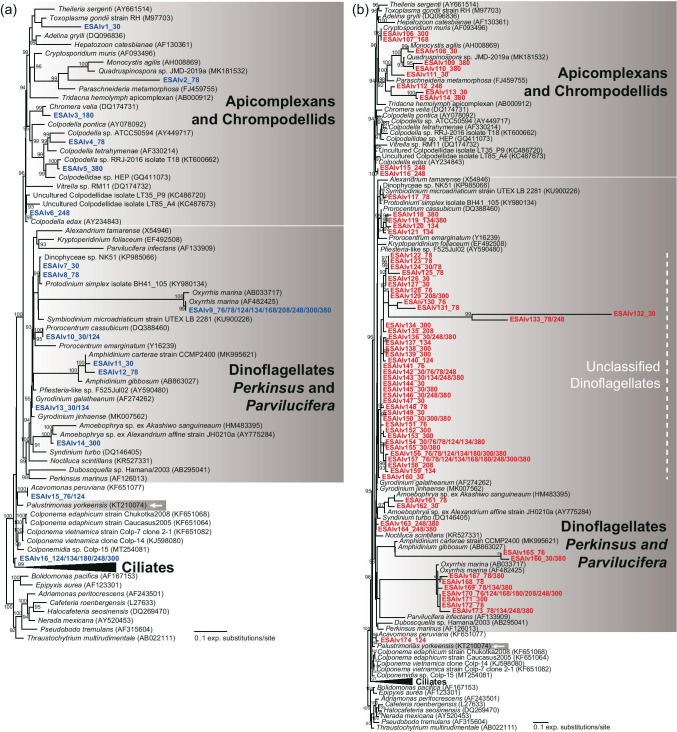
Maximum likelihood phylogenetic tree of the V4 (**a**) and V9 (**b**) regions of 18S rDNA sequences from Alveolata species except for ciliates. The sequence names from the V4 and V9 region datasets are marked in blue and red, respectively. The numerical values after the sequence name indicate the practical salinity units of the samples in which each OTU was found. White arrows represent halophilic species, which can grow best at 75 psu or higher. Bootstrap support values are shown at the nodes when > 90%

**Fig. 10:**
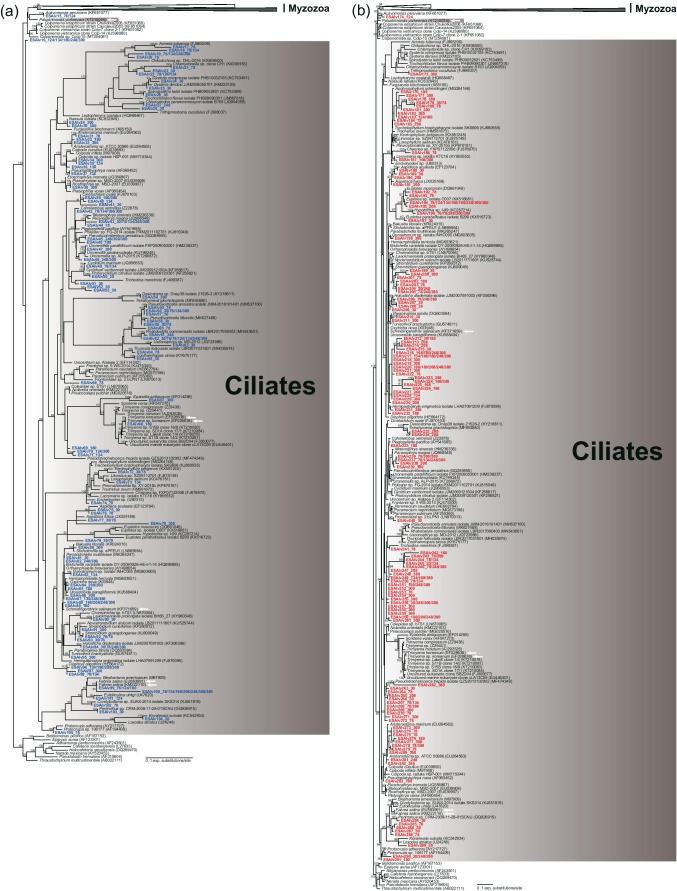
Maximum likelihood phylogenetic tree of the V4 (**a**) and V9 (**b**) regions of 18S rDNA sequences from ciliates. The sequence names from the V4 and V9 region datasets are marked in blue and red, respectively. The numerical values after the sequence name indicate the practical salinity units of the samples in which each OTU was found. White arrows represent halophilic species, which can grow best at 75 psu or higher. Bootstrap support values are shown at the nodes when > 90%

A total of 105 OTUs and 186 OTUs for the V4 and V9 regions, respectively, were assigned to the alveolate subgroups Apicomplexa (and relatives; e.g., chrompodellids), Dinoflagellates, Ciliates, and *Acavomonas* (Figs. 9 and 10). Most of the sequences belonged to ciliates (V4: 84.8%, V9: 62.9%, Fig. 10). Usually, the V4 region sequences were assigned to previously reported groups in Alveolata, while many V9 sequences were affiliated with unclassified Alveolata groups (Figs. 9 and 10).

## Discussion

Through NGS-based studies, which can provide expanded knowledge about the diversity and succession of the unexplored microbial eukaryotes in natural ecosystems, including extreme habitats [26, 31, 53, 54], several new and noteworthy observations were made in this study: 1) Most high-abundance (RA of > 5%), low-abundance (RA of 0.1–5%), and rare (RA of < 0.1%) microbial eukaryotic groups defined based on read counts and the number of OTUs exhibited a persistent distribution pattern across the salinity gradient throughout seasons and years (see below). 2) Only Discoba, which is a predominant group cultured from hypersaline habitats [1, 14, 17, 22, 51, 55, 56], could switch between the low-abundance and high-abundance groups, suggesting that it is an opportunistic (*r*-selected) group in hypersaline environments. Moreover, 3) a nonlinear inverse relationship between Alveolata and Stramenopila implies a predator–prey interaction (see below). Finally, 4) a high degree of novelty was detected in the low-abundance Amoebozoa, Cryptista, Haptista, and Rhizaria groups and the high-abundance Stramenopila group of eukaryotes, indicating that the detection of novel eukaryotes is still insufficient (see below).

### Diversity of High-Abundance, Low-Abundance, and Rare Eukaryotic Groups

Our study revealed the dominance of four high-abundance groups (RA of > 5%) in hypersaline environments, namely, Alveolata, Opisthokonta, Stramenopila, and Archaeplastida, which usually collectively represented > 90% of reads, regardless of the type of primer set, salinity and sampling time. Moreover, these four high-abundance groups usually represented > 80% of OTUs in both the V4 and V9 region datasets. OTUs at lower taxonomic ranks were mainly assigned to *Fabrea* in Alveolata, *Dunaliella* in Archaeplastida, *Artemia* in Opisthokonta, and *Halocafeteria* in Stramenopila. The four genera *Fabrea*, *Dunaliella*, *Artemia*, and *Halocafeteria* are all commonly reported as halotolerant/halophilic in diverse hypersaline habitats [1, 2, 12, 23, 52]. Our results indicate that the dominance of high-abundance groups (i.e., Alveolata, Opisthokonta, Stramenopila, and Archaeplastida) is maintained in hypersaline environments, and the genera described in high-abundance groups were restricted to hypersaline environments. Consistently, a DNA-based study (i.e., DGGE-cloning-sequencing) demonstrated that 90% of all phylotypes belonged to Archaeplastida (37%), Alveolata (30%), Stramenopila (12%), and Opisthokonta (11%) in 34 saline water environments (12.5 to 384 psu), including solar salterns, in Spain and Chile [20]. However, the genetic diversity of high-abundance biota depends on sampling location and/or physiochemical properties as well as methodology [19, 57]. The protistan diversity (except Opisthokonta) in a Portuguese solar saltern was dominated by Archaeplastida, Alveolata, and Stramenopila, collectively representing 75.6% of the V4 OTUs at 40 psu, 81.7% at 120 psu, and 94.3% at 380 psu detected by 454 pyrosequecing [19]. In deep hypersaline anoxic basins (~ 3,500 m depth), OTUs of Alveolata (72%) and Opisthokonta (22%) significantly contributed to the eukaryotic community revealed by an 18S rDNA clone library [58]. In hypersaline samples from Lake Tyrrell, Australia, containing high sulfate concentrations (15 g L^−1^) [59], halophilic *Colpodella* (Alveolata, 84.1–98.3%) and *Dunaliella* (Archaeplastida, 0.4–10.4%) were the predominant groups in summer and winter according to an 18S rDNA clone library [37]. Thus, it seems that most of the sequences identified in hypersaline environments belong to the four high-abundance groups Alveolata, Opisthokonta, Stramenopila, and Archaeplastida.

In contrast, the low-abundance and rare groups (low-abundance groups: Amoebozoa, Rhizaria, Telonemia, Cryptista, Discoba, and Haptista; rare groups: Apusomonada, Breviates, Ancyromonadida, CRuMs, and Picozoa) in hypersaline environments can be extremely resilient to salinity changes and times of sampling. In fact, the low-abundance and rare groups rarely became high-abundance groups, except for Discoba (see below). This result suggests that most of low-abundance and rare microbes are permanently distributed in hypersaline environments and display no periodic increase in abundance [25, 60]. These low-abundance and rare groups have low-range fluctuations (Fig. 3a) and somehow enable them to become low-abundance or rare groups in these extreme habitats. Strikingly, however, Discoba appears to be an opportunistic group (*r*-selected) based on read counts. Discoba, a mostly low-abundance group in this study, sometimes became a high-abundance group in the eukaryote taxonomic profiles in the V9 dataset (ranked second among eukaryotes: 28.2% at 180 psu, ranked third among eukaryotes: 7.2% at 248 psu). It is possible that abiotic factors (e.g., ion composition, salinity, and temperature) greatly influence the abundance pattern of Discoba [18, 22, 24, 55, 61]. Several studies have demonstrated that some low-abundance microbes play a role in species blooming or succession, whereas others are permanently rare or temporarily opportunistic groups [25, 62, 63]. Hence, it is likely that low-abundance or rare groups display a strict distribution pattern over time in hypersaline environments, but there are exceptional cases, such as Discoba, in such environments. Therefore, the hypersaline eukaryotic community may include permanently high-abundance, low-abundance, and rare groups and infrequently occurring opportunistic groups, as reported in prokaryotes in nonhypersaline environments [25].

The OTUs in the V9 region dataset could be assigned to more diverse groups than those in the V4 region dataset [26–28, 64]. Furthermore, unclassified eukaryotes were more represented among the V9 sequences than among the V4 sequences. This result indicates that the V9 primer set is an efficient biomarker for detecting diverse eukaryotes in hypersaline environments, similar to in nonhypersaline habitats [26]. Interestingly, numerous low-identity sequences in the V4 and V9 datasets were distributed in diverse groups. It is noteworthy that OTUs with < 90% sequence identity with the closest sequence in GenBank were frequently found in low-abundance or rare groups, consistent with previous indications that much of the taxonomic novelty in very large sequencing datasets occurs in low-abundance or rare eukaryotes [25, 63]. The proportions of novelty in the high-abundance and low-abundance groups in the V9 region dataset were 12% (185 OTUs of a total of 1,592 OTUs) and 28% (96 OTUs of a total of 337 OTUs), respectively. In Portuguese salterns, 27% of all OTUs showed < 90% sequence identity with the closest sequence in GenBank [19], and eight of 73 (i.e., 11%) 18S rDNA sequences in hypersaline habitats had < 90% sequence identity with any previously deposited sequence in GenBank based on a classic denaturing gradient gel electrophoresis approach [20]. Therefore, it seems that the novelty of eukaryotes remains high in hypersaline environments. In particular, in the low-abundance group, Amoebozoa, Cryptista, Haptista, and Rhizaria, with at least > 30 OTUs in the V9 region dataset, displayed the highest novelty (up to 46%, Table 3). In the high-abundance group, Stramenopila seemed to have the highest novelty (11.1% for V4, 21.0% for V9, Table 3). Our results provide convincing evidence for the groups with high novelty, and it can be inferred that these novel biotas may contribute greatly to the microbial food web in hypersaline habitats.

Salinity is the main factor influencing the biota in hypersaline environments, where the species diversity decreases with increasing salinity [5, 9, 65, 66]. However, the identities of other factors influencing the diversity of the biota are unknown. Here, some high-abundance species in Alveolata and Stramenopila groups were inversely coupled with each other in hypersaline environments. Moreover, the occurrence of the dominant species differed between waters with similar salinity levels (see Fig. 6). The causes of these results are unclear, and in addition to salinity, ecological interactions between some Alveolata and Stramenopila may exist. Certainly, the most abundant Alveolata members (*Fabrea salina* and *Parabistichella multilineae*: 120–220 μm in length) are larger than the dominant Stramenopila members (*Caecitellus*, *Halocafeteria*, *Nitzschia capitellata*, and *Navicula cryptotenelloides*: 2–70 μm in length) in low- and/or high-salinity waters [23, 67–70]. Considering the dominant species of Alveolata and Stramenopila and their significant inverse relationships, it is likely that larger Alveolata ciliates (*Fabrea salina* and *Parabistichella multilineae*) prey upon smaller Stramenopila bicosoecids (*Caecitellus* and *Halocafeteria*) and diatoms (*Nitzschia capitellata* and *Navicula cryptotenelloides*) [68, 71]. Further studies may shed light on the ecological interactions between relatively larger Alveolata and smaller Stramenopila in hypersaline environments.

### Comparison with Cultivation-Based and Microscopy-Based Studies

Hypersaline environments are excellent systems for examining species succession, adaptation, evolution, novelty, diversity, and activity. Several sequencing-based and microscopy-based studies have reported that the eukaryotic community differed substantially in salt ponds with different salinities [19, 20, 65, 66, 72–74]. In our study, the dominant *Dunaliella*-like and *Tetraselmis*-like sequences in Archaeplastida were mainly detected at > 100 psu and at < 100 psu, respectively (Fig. 4), similar to the findings of previous studies that *Dunaliella* grew at > 100 psu and *Tetraselmis* could grow from 30 to 110 psu [75, 76]. Furthermore, the dominant sequences related to *Navicula*, *Nitzschia*, *Caecitellus*, and *Halocafeteria* in Stramenopila were mostly detected at > 150 psu, whereas *Fabrea salina*-like sequences in Alveolata were distributed from 76 to 380 psu (Fig. 4). The genera *Halocafeteria* and *Fabrea* could thrive in a broad range of 75 to 300 psu (see below) [23, 77]. The other three stramenopiles could tolerate up to 180 psu [44, 78]. Overall, the source salinity regime of the OTUs detected in this study usually coincided with the salinity range suitable for the growth of closely related halotolerant/halophilic species.

Heterolobosea is known as the predominant group cultured from hypersaline habitats, but heterolobosean sequences have rarely been detected in 18S rRNA/DNA clone libraries from field samples [12, 20, 37, 58, 79, 80]. Here, the high occurrence of classified or unclassified heterolobosean sequences is an outstanding feature of the V9 region dataset. For the first time, two OTUs related to *Percolomonas*, which was previously reported from saturated brines, were detected in four samples (168–380 psu) by a culture-independent approach. Furthermore, many unclassified OTUs were scattered across heterolobosean groups previously reported as halophilic, such as Tulamoebidae, *Aurem*, *Euplaesiobystra*, and *Pharyngomonas* [1, 14, 17, 22, 51, 55, 56]. The family Tulamoebidae, including *Tulamoeba*, *Pleurostomum*, and *Aurem,* is regarded as unique adaptative radiation of halophilic eukaryotes [22, 51]. Moreover, the species inventory of other halophilic/halotolerant groups in Heterolobosea was further expanded. Several OTUs related to typical freshwater or marine heterolobosean species (e.g., *Naegleria* and *Heteramoeba*) were recovered from high-salinity waters, and they may form cysts to remain viable [18, 81, 82]. Similar to our results, Post et al. [74] reported that *Neagleria* spp. and *Heteramoeba* sp. were detected in water with salinities up to saturated brine and 210 psu, respectively, in Hutt Lagoon, Australia. Alternatively, they may be present as dead cells. In our study, the abundance of an OTU (ESHet11_168/208/248/300, Fig. 7) closely related to freshwater *Naegleria* greatly decreased with increasing salinity from 168 psu (2,995 reads) to 300 psu (28 reads). Thus, our results suggest that the detection of heterolobosean species depends strongly on the primer set used (and/or sampling location) and that the heterolobosean species in hypersaline environments are more diverse than previously realized [26].

Among stramenopiles, *Halocafeteria,* which grows at 100–363 psu, is a common culturable bicosoecid in hypersaline habitats worldwide [12, 23]. In addition, several undescribed halotolerant species in Placididea (MESS13, ME5, MESS14, and ME8) have been cultured [44]. Interestingly, in addition to the OTUs related to previously reported species, many unknown stramenopile OTUs were also found. This result suggests that previously undiscovered species are "hidden" in hypersaline habitats. Both the V4 and V9 datasets included a wide diversity of sequences with phylogenetic affinities to Bicosoecida. In addition, however, the V9 primer set also included MAST-3 and unclassified halotolerant stramenopiles, which were undetected with the V4 primer set. Both the MAST-3 and MAST-4 clades are considered bacterivorous and widely distributed in oceans [83, 84]. Thus, a large number of undiscovered stramenopiles, such as novel clades within MAST-3, are essential to maintain microbial food webs in hypersaline environments. In addition, the majority of stramenopiles in hypersaline environments have greater phylogenetic complexity than previously thought.

The V9 region dataset had a much-improved phylogenetic affinity for Alveolata compared with that of the V4 region dataset, similar to the results for Stramenopila and Heterolobosea. Due to the numerous rDNA copy numbers in ciliates, the relative abundance of ciliates might be overestimated [85, 86]. The fractions of ciliates among the total read counts at 40–120 psu in a solar saltern were 17–19 times higher than those of protists directly examined with the fluorescent in situ hybridization method [19]. The numbers of dominant ciliate-related sequences in Alveolata were 89 OTUs for V4 and 117 OTUs for V9, comparable to the 86 ciliate-affiliated OTUs in the V4 region pyrosequencing dataset [19]. A few halophilic/halotolerant ciliates were successfully cultured: two halophiles, *Trimyema koreanum* and *Platynematum salinarum*, which could grow at 140–300 psu [11, 50]; two halotolerant species, *Schmidingerothrix extraordinaria* and *Euplotes qatarensis*, that grew at 10–110 psu [87, 88]; and another halotolerant species, *Fabrea salina*, that could grow at 58–311 psu [77, 89]. Furthermore, *Palustrimonas yorkeensis* and *Colpodella* spp. may represent halophilic alveolates [12, 13, 37, 74, 90]. Hence, diverse alveolates were also detected in hypersaline environments, and it is striking that several unclassified Alveolata clades were only found in the V9 region dataset (Figs. 9 and 10). Our findings suggest that a diverse assemblage of unclassified alveolates is an essential group within the eukaryotic community in hypersaline environments.

## Conclusions

In this study, the high-abundance groups Alveolata, Stramenopila, Archaeplastida, and Opisthokonta remained the dominant groups along salinity gradients across different seasons and years, but the most abundant group varied. In contrast, most low-abundance or rare groups remained small (< 5% read counts) and appeared to have a strictly suppressed distribution in the hypersaline eukaryotic community, irrespective of season and year. Thus, most of the high-abundance, low-abundance, and rare groups display no periodic increase/decrease in abundance in this unique environment, and the ecological stability of microbial eukaryotes appears to persist along the salinity gradient during the field surveys. The inverse relationship of some high-abundance species in Alveolata and Stramenopila groups might reflect predator–prey ecological interactions (larger Alveolata *vs.* smaller Stramenopila). As a result, the microbial eukaryotic community displays greater phylogenetic complexity than previously realized. Remarkably, the level of taxonomic novelty with < 90% sequence identity was greater in low-abundance and rare eukaryote groups, suggesting that many new findings have yet to be made. Diverse "hidden" groups may additionally contribute to the eukaryotic community in this extreme habitat.

## Data Availability

The sequences were deposited in NCBI GenBank under accession numbers MZ209813-MZ296921, MZ296938-MZ297139, and MZ299397-MZ299685 for the V4 region and MZ297140-MZ297237 and MZ299686-MZ300855 for the V9 region. Corresponding sample descriptions are accessible through BioProject PRJNA732544.
